# Assessing the impact of transitions from centralised to decentralised water solutions on existing infrastructures – Integrated city-scale analysis with VIBe

**DOI:** 10.1016/j.watres.2013.10.038

**Published:** 2013-12-15

**Authors:** Robert Sitzenfrei, Michael Möderl, Wolfgang Rauch

**Affiliations:** Unit of Environmental Engineering, Institute of Infrastructure, University of Innsbruck, Technikerstr. 13, 6020 Innsbruck,Austria

**Keywords:** City-scale integrated analysis, Hydraulic simulation, Virtual Infrastructure Benchmarking, Decentralised, Sustainable urban drainage

## Abstract

Traditional urban water management relies on central organised infrastructure, the most important being the drainage network and the water distribution network. To meet upcoming challenges such as climate change, the rapid growth and shrinking of cities and water scarcity, water infrastructure needs to be more flexible, adaptable and sustainable (e.g., sustainable urban drainage systems, SUDS; water sensitive urban design, WSUD; low impact development, LID; best management practice, BMP). The common feature of all solutions is the push from a central solution to a decentralised solution in urban water management. This approach opens up a variety of technical and socio-economic issues, but until now, a comprehensive assessment of the impact has not been made. This absence is most likely attributable to the lack of case studies, and the availability of adequate models is usually limited because of the time- and cost-intensive preparation phase. Thus, the results of the analysis are based on a few cases and can hardly be transferred to other boundary conditions. VIBe (Virtual Infrastructure Benchmarking) is a tool for the stochastic generation of urban water systems at the city scale for case study research. With the generated data sets, an integrated city-scale analysis can be performed. With this approach, we are able to draw conclusions regarding the technical effect of the transition from existing central to decentralised urban water systems. In addition, it is shown how virtual data sets can assist with the model building process. A simple model to predict the shear stress performance due to changes in dry weather flow production is developed and tested.

## Highlights

• Integrated analysis of transition from central to decentralised urban water systems.
• Innovative stochastic generation of city-scale case studies successfully applied.
• Coupling water distribution and urban drainage simulations via population.
• Analysis of 81 test cases to identify stable operation ranges for water systems.
• Development and application of a model to predict shear stress performance, *R*^2^ = 0.99.

## Graphical abstract

**Figure:**
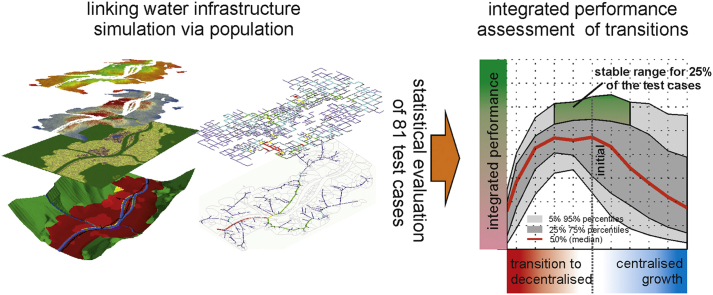

## 1 Introduction

Traditional urban water management relies on central organised infrastructure, the most important being the drainage network and the water distribution network. To meet new challenges, such as climate change and changes in the population and land use (growth as well as shrinkage in the cities), it is commonly agreed that water infrastructure needs to be more flexible, adaptable and sustainable (e.g., Brown et al., 2009, Domènech and Saurí, 2010). These efforts towards increased sustainability are denoted sustainable urban drainage systems, SUDS; water sensitive urban design, WSUD; low impact development, LID; and best management practice, BMP (e.g., Ole et al., 2012). The common feature of all solutions is the push from a central solution to a decentralised solution in urban water management. This transition opens up a variety of technical and socio-economic issues, but until now, a comprehensive assessment of the impact was missing. This absence is mostly attributable to the lack of case studies, and the availability of adequate models is usually limited because of the time- and cost-intensive preparation phase. This preparation includes, among other things, data collection, digitalisation, model construction and calibration (Refsgaard and Henriksen, 2004). However, findings based on a single or just a few case studies are very case specific, and the conclusions can hardly be generalised. In this paper, we present an integrated city-scale analysis based on virtual generated urban water systems. With this approach, we are able to form conclusions about the technical effect of the transition from central to decentralised urban water systems.

Virtual case studies have been created and used in various studies (e.g., Achleitner et al., 2007, Borsanyi et al., 2008, Butler and Schütze, 2005, De Toffol et al., 2007, Lau et al., 2002, Meirlaen et al., 2001, Schütze et al., 2002, Rauch et al., 2003) to allow for detailed investigations, even if data are not available. These studies' properties are intended to resemble real-world characteristics, and the case studies are thus referred to as either synthetic, artificial, (semi-)hypothetical or virtual (Sitzenfrei, 2010).

In the field of water distribution system analysis (WDSA), the three example networks provided with the hydraulic solver EPANET2 (Rossman, 2000) have been used in numerous investigations as benchmark systems. Such benchmark systems serve to test the coherence of numerical solvers, as well as to allow for the comparison of methods and solutions. Other established benchmark systems of WDSA are the New York Tunnels system, the Two-Loop system, the Anytown network and the Hanoi network (Sitzenfrei et al., 2013). In the field of urban drainage modelling (UDM), the use of benchmark systems is less established compared to WDSA. However, several benchmark systems were introduced (e.g., Schilling, 1989, Schütze et al., 1999) to test new approaches (e.g., Rauch and Harremoës, 1999, Zacharof et al., 2004).

Because of the lack of data regarding the accurate description of ageing and deterioration of urban drainage systems, a network condition simulator (NetCoS) was developed and used in Scheidegger et al. (2011) and Scheidegger and Maurer (2012), respectively. Blumensaat et al. (2012) developed a model for generating sewer models under minimum data requirements, which are to be seen as semi-synthetic case studies. Furthermore, stochastic approaches were developed for the algorithmic generation of conceptual, simplified network models (water distribution and combined sewer systems) based on the variation of layout and size, among other network characteristics (Möderl et al., 2007, Möderl et al., 2009, Möderl et al., 2011, Trifunovic et al., 2012). Each of these approaches provides an interface to a hydraulic solver (for WDSA: EPANET2, Rossman, 2000; for UDM: SWMM, Rossman, 2004, Gironás et al., 2010) to analyse the hydraulic performance of the generated networks. However, all of the approaches mentioned above neglect the urban structures (population densities, land use) in the network design and only add the “city layout” after the network generation.

However, for an integrated assessment of transition effects, considering the dynamics at the city scale is essential. In this paper, a novel concept for the generation of virtual urban water systems is presented. In VIBe (Virtual Infrastructure Benchmarking), the urban structure (“virtual city” including population densities, land use and topography) is generated first. Second, the infrastructure networks (including interfaces to hydraulic simulation software) are created according to the state-of-the-art design rules and meet the requirements of the “virtual city”. Therefore, the generated infrastructure networks can be spatially linked via the population.

VIBe is based on a number of sub-modules that have been described earlier, i.e., urban structure generation (Sitzenfrei et al., 2010a), sewer generation (Urich et al., 2010) and the water distribution system generation (Sitzenfrei et al., 2010c). In this work, it is shown, how these approaches can be coupled for the integrated analysis of the urban water system. This approach allows the transition of present systems towards decentralised implementations to be analysed (e.g., biofiltration systems, rainwater harvesting, water reuse and infiltration) (e.g., Le Coustumer et al., 2012, Ward et al., 2012, Moore et al., 2012, Dong et al., 2012, Peter-Varbanets et al., 2009, Barton and Argue, 2009) with respect to the impact on the hydraulics, water quality and emissions performance of the existing centralised systems. In addition, it is shown how virtual data can assist in model building. Therefore, a simple model to predict shear stress performance due to changes in dry weather flow production is developed and tested.

## 2 Methods

In this section, the modelling concepts of the VIBe approach are described (Section 2.1). Further, the generation process of city-scale case studies (Section 2.2) is discussed. How the urban structures (Section 2.2.1) (Sitzenfrei et al., 2010a), the sewer systems (Section 2.2.2) (Urich et al., 2010) and the water distribution systems (Section 2.2.3) (Sitzenfrei et al., 2010c) are generated with the VIBe approach is outlined.

### 2.1 Modelling concepts

**Fig. 1:**
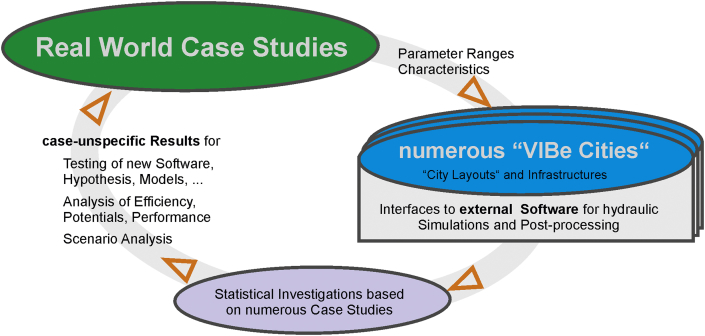
Modelling concept of VIBe.

VIBe algorithmically generates numerous case studies of urban water systems at the city scale, including infrastructure networks. Therefore, numerous scenarios with different characteristics can be created for further statistical investigations. To provide a hydraulic performance analysis for the generated infrastructure systems, interfaces to external hydraulic simulation software are implemented in the VIBe approach. This allows for generic findings from the statistical evaluations of the results. This helps to identify system coherences, to determine potentials and to enhance the understanding of generated and real-world systems (see Fig. 1). Furthermore, new software, hypotheses or modelling approaches can be tested, e.g., the potential of real time control, urine separation, rainwater infiltration, optimisation algorithms, parallel modelling algorithms, transition of water savings scenarios.

### 2.2 Generation procedure

The required data are identified from the literature and from real-world case studies, and the parameter ranges are subsequently determined. The “city layout” is created with the urban structure module (US-Module) and by stochastic sampling within the parameter ranges. With predefined topographic boundary conditions (e.g., location in an Alpine valley or size of urban agglomeration), the US-Module generates the digital description of a virtual city (see Section 2.2.1).

**Fig. 2:**
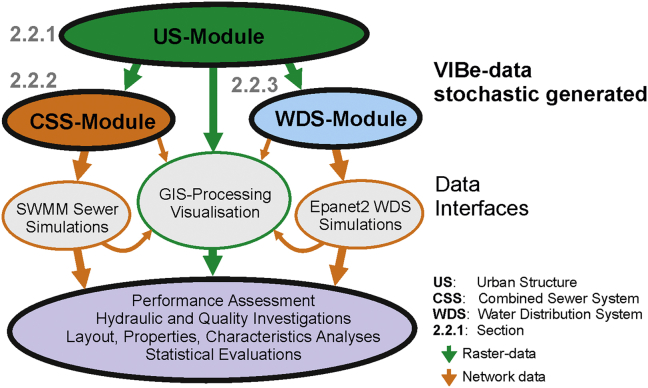
Concept of the generation process in VIBe.

The infrastructure modules generate water networks based on the virtual “city layouts” (see Fig. 2). Hence, in the generation process, the “city layout” is created first, represented as GIS data (raster data).

Subsequently, the current implemented infrastructure modules, both the combined sewer system module (CSS-Module, see Section 2.2.2) and the water distribution system module (WDS-Module, see Section 2.2.3) create (with stochastic layout variations) infrastructure systems according to state-of-the-art design rules and stochastic under- or overdesign. For the US-Module, interfaces for GIS-processing and interfaces to external hydraulic modelling tools are supplied. The concept introduced in VIBe has been developed for urban water systems but can be applied to other urban infrastructure (e.g., energy supply, gas network and district heating).

#### 2.2.1 Urban structure (“city”) module (US-Module)

**Fig. 3:**
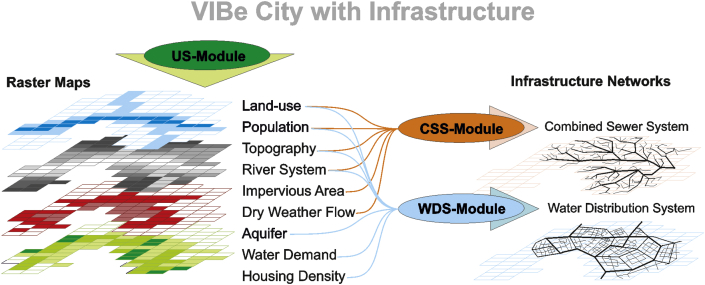
Creation of a VIBe city.

The US-Module creates entire cities but without water infrastructure. The generated model builds upon the properties and characteristics of real topography and river systems, and in this study, the model is based on an analysis of an Alpine region. Such a region is characterised by a flat valley floor and a river which meanders (Sitzenfrei et al., 2010a). The US-Module provides all required data for the infrastructure modules with characteristics that are comparable with Alpine real cities but the approach can also be enhanced for other topographies. Furthermore, GIS maps required for water infrastructure systems, such as impervious area, dry weather flow, water demand, and aquifers, are provided by the US-Module (see also Fig. 3) and can be exported via an interface to GIS-processing software.

#### 2.2.2 Combined sewer system module (CSS-Module)

**Fig. 4:**
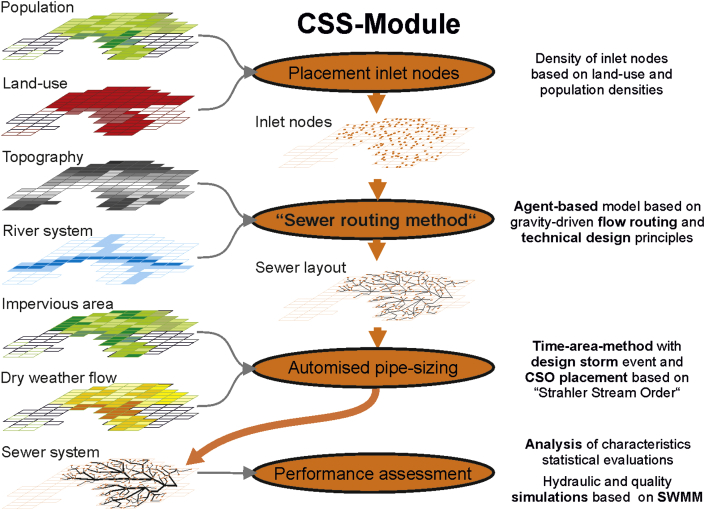
Procedural method of the CSS-Module.

The CSS-Module in VIBe generates combined sewer systems based on GIS data created either with the US-Module (see Figs. 3 and 4) or real-world data. This module generates layouts of sewer systems meeting the requirements of the previously defined cities based on the state-of-the-art design rule and stochastic variations. In addition, the generated sewer systems are pipe-sized and exported as an SWMM (Rossman, 2004) input file.

The required inlet nodes to the sewer system are placed based on the GIS maps for population density and land use (Fig. 4, placement of inlet nodes). The inlet nodes of the sewer systems are automatically connected to a sewer layout, which drains the urban area to a waste water treatment plant (WWTP). To that, a “sewer routing method” has been developed which takes into account (gravity driven) flow routing and technical design principles for the layout of combined sewer systems (e.g., vertical alignment, WWTP, manhole spacing for direction changes, minimal slope and culverts for river-crossing). This creation process of the layout of the combined sewer system is applying agent-based modelling techniques. The movement paths of the agents represent the layout of the sewer pipes result in a fully branched network (see Fig. 4).

**Fig. 5:**
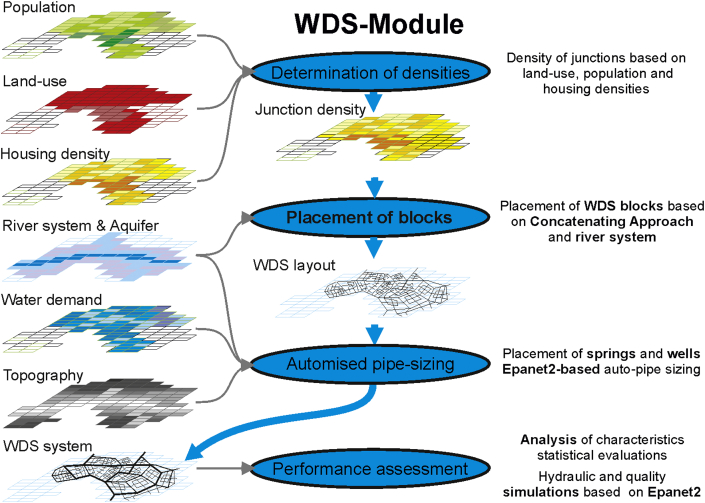
Procedural method of the WDS-Module.

Subsequently, the sewer networks are algorithmically pipe-sized based on the time-area method (Butler and Davies, 2004) and a design-storm event (taking into account the spatial distributed data for impervious area and amount of dry weather flow; see Fig. 4). In these combined sewer networks, besides nodes and junctions, also storage units, weirs and combined sewer overflows are regarded. The resulting combined sewer systems are exported to the hydraulic solver SWMM (see Fig. 5). A detailed discussion of the entire process and its limitations can be obtained in Urich et al. (2010).

#### 2.2.3 Water distribution module (WDS-Module)

With the WDS-Module of VIBe (Sitzenfrei et al., 2010c), water distribution systems (WDS) are generated based on GIS data from the US-Module (see Figs. 3 and 5) or real-world data. The generation process is based on maps for topography, land use, population densities, housing densities, river systems, aquifers and water demand.

Based on the GIS maps for population density, land use and housing density, a map for the required junction densities is created (Fig. 5, placement of inlet nodes). The generation process of WDS implemented in VIBe follows the idea of complex network structures composed of recurring network motifs (Milo et al., 2002). Different network motifs (WDS-blocks) are designed by means of a graph theory based approach. The diverging WDS-blocks represent different redundancies and arrangements of pipe connections (e.g., branched or looped blocks) and different housing densities (assumed to correspond with network junction densities). For the generation process, a database of different WDS-blocks has been developed, tested and compared with real-world network structures (Sitzenfrei et al., 2013). With this database and the maps for housing and population densities, entire complex WDS can be composed by selecting WDS-blocks from the database that meet the requirements of the GIS data in the “city layout” and concatenating those WDS-blocks to the entire WDS. Subsequently, the water demand and elevation are added spatially distributed to junctions in the generated WDS. Finally, water sources are added to the generated WDS. Depending on the topographic and topological boundary conditions, hillside springs or groundwater wells are added. Therefore, gravity driven supply as well as pumped systems can be generated. Components for pressure management (like pressure reducing valves) are at the current state of development not regarded.

Next, the diameters of the WDS are designed based on the economic flow velocity. Using an iterative approach (Möderl et al., 2007), the flow velocities for the entire WDS are determined with the EPANET2 hydraulic solver. The diameters are then incrementally augmented as long as the actual flow velocity exceeds the defined economic one. For performance evaluation, an interface to the hydraulic solver EPANET2 is implemented.

## 3 Integrated scenario analysis of water infrastructure

Fratini et al. (2012) noted that for the transition of traditional water infrastructure towards sustainability, it is important to be more engaged with other disciplines (e.g., social sciences, urban planning and architecture). The VIBe approach allows for an integrated scenario analysis that takes into account the entire urban water infrastructure and the structure of the city. The GIS information of the population and water demand is spatially linked to the water infrastructure models (WDS model and UD model). Therefore, changes in the population densities and the water demand can directly be linked to the WDS and UD models, respectively.

### 3.1 Coupling urban water infrastructure

The basis for all further calculations is the total daily average water demand, *Q*_d,m,t_, determined with the spatial distribution of the population (population equivalents (PE)). From that, the relevant water flows and multiplication factors (i.e., prospective demand factor *f*_p_, daily peaking factor *f*_d,max_, hourly peaking factor *f*_h,max_, maximum dry weather flow *f*_DWF,max_) relevant for the design of systems of the investigated size (above 50,000 PE) and performance assessment (ÖNORM, 2002, ÖWAV-RB 11, 2009, ÖWAV-RB 19, 1987) can be determined:

For the different design and performance assessment scenarios (see also Sections 3.2, 4), the water flows in the hydraulic modelling processes are determined according to Table 1.

**Table 1:** Assessment and design flows for WDS and UD.

Description	Variable name	Factor(s)	Composition	Application (see also Section 3.2)
Total current daily average water demand	*Q*_d,m,t_	–	–	Basis for further calculations
Water losses	*Q*_WL_	*f*_WL_ = 0.08–0.12	*Q*_d,m,t_ × *f*_WL_	Fraction of *Q*_d,m_
Current daily average water demand	*Q*_d,m_	–	*Q*_d,m,t_ − *Q*_WL_	WDS: current water quality performance
Future hourly peak flow of maximum day	*Q*_h,max,max,f_	*f*_p_ = 1.3 *f*_d,max_ = 1.4 *f*_h,max_ = 1.44	*Q*_d,m_ × *f*_p_ × *f*_d,max_ × *f*_h,max_ + *Q*_WL_	WDS: network design
Current hourly peak flow of maximum day demand	*Q*_h,max,max_	*f*_d,max_ = 1.4 *f*_h,max_ = 1.44	*Q*_d,m_ × *f*_d,max_ × *f*_h,max_ + *Q*_WL_	WDS: current hydraulic performance
Sewer infiltration water	*Q*_i_		0.75–1.25 l/(1000 PE d)	UD: fraction of *Q*_DWF,hyd_
Current DWF for hydraulic performance (maximum DWF)	*Q*_DWF,hyd_	*f*_DWF,max_ = 1.2	*Q*_d,m_ × *f*_DWF,max_ + *Q*_i_	UD: with rain weather flow for design and CSO and flooding assessment
Current DWF for shear stress performance (minimum DWF)	*Q*_DWF,τ_		120–150 l/(PE d)	UD: without rain for shear stress performance

### 3.2 Performance assessment

For the performance assessment, the generated networks are simulated with the software tools EPANET2 (water distribution system) and SWMM5 (urban drainage) by applying the water demands and dry weather flows according to Table 1. In the WDS model, the pressure distribution in hydraulic peak flow situations is assessed by means of a steady state simulation (only one time step). For the water quality analysis, the residence time of the water in the pipe network is investigated. Therefore, a simulation time of 50 h is used (series of steady state simulations).

For the UD model, a design-storm event of type EULER II (ATV-A 118E, 2006) with a return period of 5 years and a duration of 2 h is used. The simulation time for the rain weather simulation is 24 h.

For the performance assessment, the following performance indicators normalised between 0 and 1 are used:

• CSO performance (UD CSO): ratio between the total volume of the surface runoff that is treated at the waste water treatment plant and the total surface runoff.
• Flooding performance (UD flooding): 1 minus the maximum ponded volume over all nodes divided by the total rainfall runoff.
• Bed shear stress performance (UD shear stress): the bed shear stress is calculated according to the Austrian standard ÖWAV-RB 11 (2009) for each pipe *i* with Equation 1. A bed shear stress of minimum 1 N/m^2^ indicates sufficient performance. Therefore, all pipes with a stress value above or equal to 1 N/m^2^ are assessed as 1. For pipes below that threshold, the actual bed shear stress (hence between 0 and 1) is used for evaluation. The sum of all values (between 0 and 1) divided by the number of pipes results in the performance indicator used for the bed shear stress. In simplified terms, this performance indicator not only expresses the occurrence of sewer sedimentation but is representative for all issues related to low flow conditions, such as sewer gas production or corrosion.

(1) $$\tau_{i}=\rho \cdot g\cdot S_{i}\cdot D_{H,p,i}\left(N/m^{2}\right)$$

*τ*_*i*_, shear stress for pipe *i* (N/m^2^); *ρ*, density of water 1000 (kg/m^3^); *g*, gravitational acceleration 9.81 (m/s^2^); *S*_*i*_, slope of pipe *i*; *D*_*H*,*p*,*i*_, hydraulic radius for partial filling for pipe *i* (m).

• Hydraulic performance (WDS hydraulic): for the hydraulic performance evaluation in the WDS model, the pressures in the junctions above or below the specified thresholds for Alpine systems (40 or 100 m, respectively) are assessed as 0. Between these two thresholds, a junction is assessed as 1. The sum over all junctions weighted with the demand divided by the number of junctions and the total demand results in the performance indicator for the hydraulic performance in the WDS.
• Water quality performance (WDS quality): for water quality, the water age (in this study, the travel time in the pipe network) is used as the indicator. Bacterial growth or other quality issues can be related to that indicator. If the travel time from the source to the demand nodes is lower than the threshold of 24 h, it is assessed as 1. If it is above this threshold, it is assessed as 0. The sum over all junctions weighted with the demand divided by the number of junctions and the total demand results in the performance indicator for the quality performance.
• Integrated performance: for an integrated performance assessment of both the water distribution and the urban drainage system, an overall performance as a product of all the single performance indicators described above is introduced.

For each performance indicator, a value of 1 indicates excellent performance and a value of 0 indicates total failure of the system (in terms of the used threshold values).

## 4 Definitions of scenarios and case studies

With the presented VIBe approach, 80 virtual Alpine cities (populations between 70,000 and 170,000) including the water distribution system and urban drainage system are generated. In addition, the real-world city Innsbruck in Austria (population 121,000) is analysed. For each of the generated city-scale case studies, different scenarios for changes in the population, water demand and amount of produced dry weather flow are investigated based on hydraulic simulations. These changes can represent different transition scenarios from centralised to decentralised water infrastructure. Although a time dependent change of the layout of water networks (e.g., network expansion, rehabilitation measures) is neglected, different levels of decentralisation (scenarios defined in the following) can represent such a transition over time. Such changes can be very fast (implementation in a few years), but can also be applicable to represent stepwise changes over a longer time period. For the latter, it must be assumed that the layout of the water networks remain static over the time horizon. Despite this limitation, the procedure allows to assess to what extent a transition to decentralised water solutions can be implemented before the existing centralised systems are pushed to their limit.

In this study, the assumption is made that all changes are uniformly distributed in the entire city. Although reductions may concentrate in specific areas and therefore cause specific local problems in real-world cases, this aspect is no regarded here for simplicity. However, disregarding such aspects does not change the presented approach. More detailed information about the change in the population and land use could be obtained and implemented by applying an urban development model to determine possible future conditions (Sitzenfrei et al., 2010b). To investigate possible transitions and unknown future developments systematically, the following scenarios are defined:

• Reduction scenarios: decrease of the population and reduction in the potable water demand per capita due to, for instance, water saving measures, decentralised water supply and reuse. Therefore, reduction rates can be applied to the daily average potable water demand, while water losses are kept constant (between 8% and 12% of the average potable water demand). This also impacts the produced dry weather flow, and the impervious area is assumed to remain as is. The five defined scenarios are a reduction to 80%, 60%, 40%, 20% and 10% of the initial water demand and dry weather flow production.
• Increase scenarios: increase of the population and increase in the water demand per capita. This increase is applied to the water demand and dry weather flow production, respectively, while the water losses are kept constant. In addition, a linear regression between the change in the population density and imperviousness is assumed (Chabaeva et al., 2004; impervious area (%) = population density (m^2^ × 10^4^) × 0.492 + 16.732). The five defined scenarios are increases of 20%, 40%, 60%, 80% and 100% of the initial water demand and increase of the impervious area calculated with the linear regression mentioned above.

Including the assessment of the initial situation, 11 variations for the performance assessment are evaluated for each of the 80 virtual case studies and the real-world case study Innsbruck. This results in a total of 891 investigated city-scale scenarios.

### 4.1 Case study water infrastructure Innsbruck

The Alpine city has approximately 121,000 inhabitants. The centralised potable water system is supplied mainly by one hillside spring, and water treatment is not required. The height difference between the source and most of the supply area is 100 m or more. Therefore, the regular supply is completely driven by gravity. For emergency supply, groundwater wells are installed. The average annual water demand is approximately 11.7 million m^3^, and the hourly peak demand on a maximum day is approximately 1000 l/s. The hydraulic model (EPANET2) consists of 7128 junctions and 7599 pipes.

A combined sewer system drains the urban area to a centralised waste water treatment plant. In the UD system of Innsbruck, dry weather inflow from outlying conurbation areas is fed in. To determine the spatial distribution of the population for dry weather simulations, the approximate spatial distribution derived from the water demand (WDS model) is used. The hydraulic model (SWMM5) consists of 182 subcatchments, 247 junction nodes and 275 conduit links.

## 5 Results

### 5.1 Integrated generation process – VIBe city

**Fig. 6:**
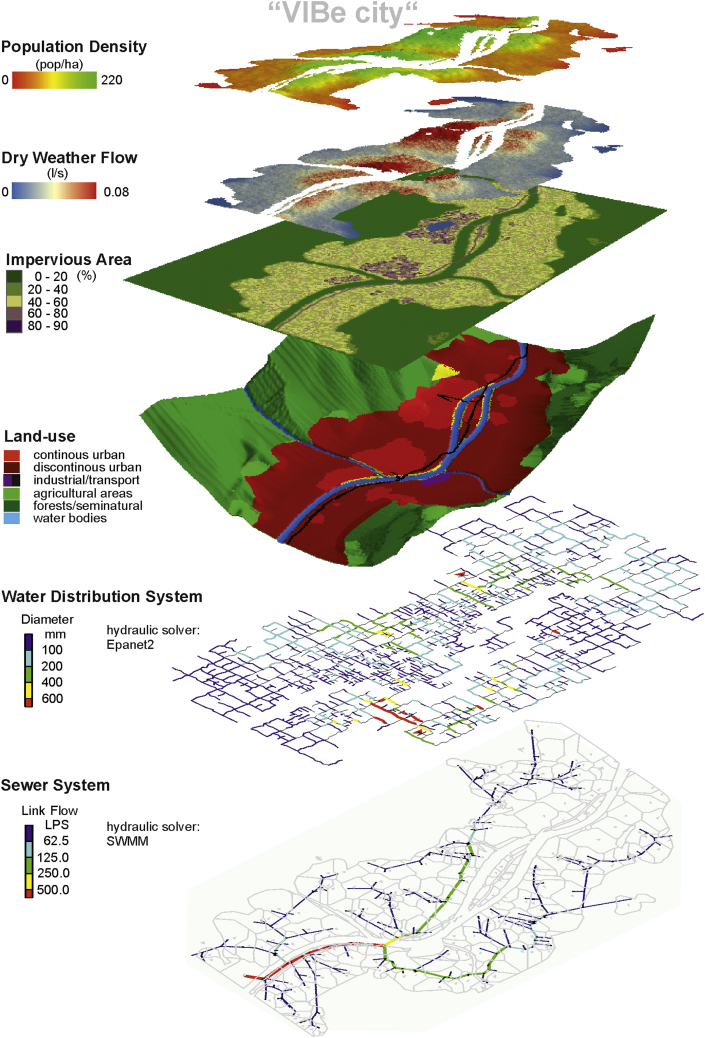
City generated with VIBe.

In Fig. 6, an example virtual city is visualised including the GIS data for population density, dry weather flow production, impervious area and land use classes. The hydraulic models of the water infrastructures are also shown.

**Fig. 7:**
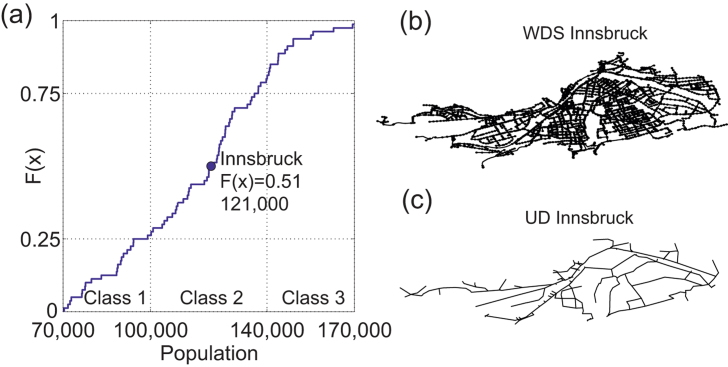
Cumulative distribution function of the generated VIBe cities with class definition (a), water distribution (b) and drainage system (c) of the real case study Innsbruck.

In Fig. 7(a), the cumulative distribution function of the population of the 80 generated Alpine cities is shown. The size of Innsbruck is approximately the median value of the generated systems (*F*(*x*) = 0.51). Three size classes are defined: (1) population below 100,000 (minimum 70,000) – class 1, about a fourth of the systems; (2) population between 100,000 and 140,000 – class 2, about half of the systems including Innsbruck; and (3) population over 140,000 (maximum 170,000) – class 3, about a fourth of the systems.

### 5.2 Impact of transitions from centralised to decentralised water infrastructures

**Fig. 8:**
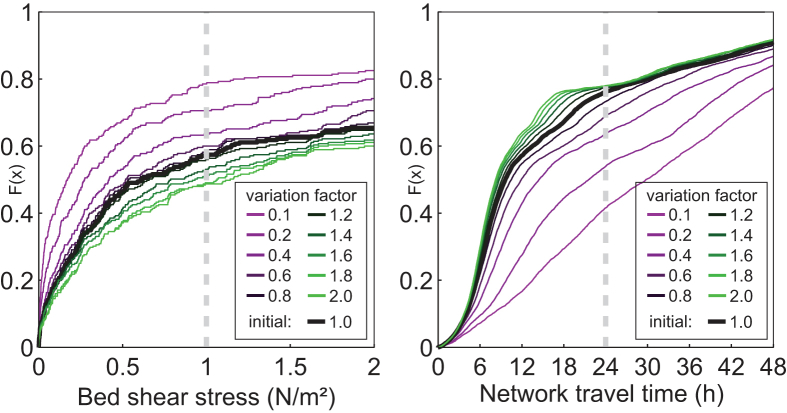
Cumulative distribution function and the impact of the variation factors on the shear stresses in the urban drainage system of Innsbruck (left) and the water age in the water distribution system of Innsbruck (right).

Fig. 8 shows the impact of the reduction and increase scenarios (expressed as variation factors) on the performance of the Innsbruck water networks. The graph on the left in Fig. 8 shows the impact for the bed shear stress as a cumulative distribution function for all pipes, and the graph on the right in Fig. 8 shows the network travel time in the WDS model. The shear stress evaluations are based on the water height calculations with SWMM5 for dry weather flow. The network travel time is a result of the EPANET2 simulations. The thick black line shows the baseline values. The dashed grey line shows the threshold values used for the performance evaluations (for the performance indicators, see Section 3.2).

For the bed shear stress, it can be observed that almost 60% of the values are below the threshold of 1 N/m^2^. An increase in the dry weather flow production does not change this picture substantially, whereas a decrease of the variation factor to 0.1 raises the percentage of values below 1 N/m^2^ to approximately 80%. For the network travel time in the WDS model, approximately 75% of the water ages in the junctions are below the threshold value of 24 h for the initial system. An increase in the water demand has less impact (shift in percentages *F*(*x*)) than a decrease.

**Fig. 9:**
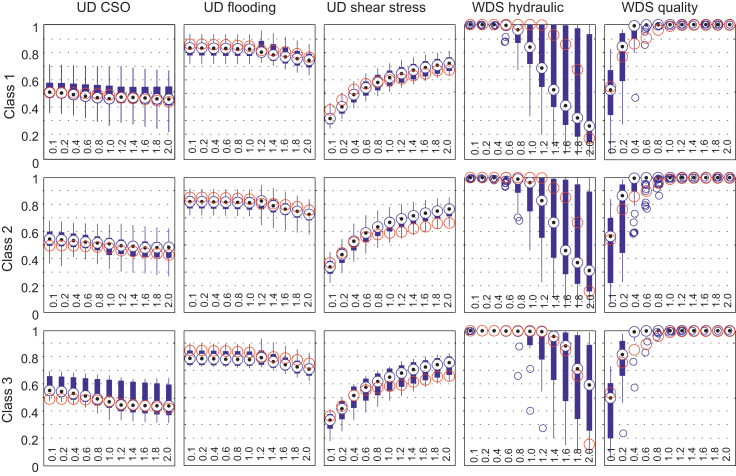
Boxplots of the performance indicators for the three different size classes.

In Fig. 9, the evaluations of all generated systems are shown aggregated to the normalised five performance indicators (five columns: UD CSO, UD flooding, UD shear stress, WDS hydraulic, WDS quality) described before. For each size class (three rows: Class 1–Class 3), a row of boxplots is shown. In each of the 15 boxes, there are 11 boxplots (for the initial, the increase and the reduction scenarios). The dots in the circles indicate the median values of the virtual systems in that class for the performance indicator and the particular variation. In addition, the performance of the real-world case study Innsbruck is shown with a circle in each boxplot.

### 5.3 Stability of the water networks

For the UD CSO performance, reductions in the dry weather flow have only a marginal impact on the performance. Thus, with regard to the transition from centralised to decentralised urban water solutions, the impact on the combined sewer system is marginal in terms of the CSO efficiency because this is mainly driven by rain weather flow. For 75% of the investigated systems, the maximum reductions in the CSO efficiency are smaller than −10%. For the case study Innsbruck, the maximum reduction is −10.9%.

In terms of the UD flooding performance, a dry weather flow reduction also only has a marginal impact on the performance. Again, per definition, this performance is mainly driven by rain weather flow. Thus, for a dry weather increase and for additional impervious area, the flooding efficiency decreases. However, the maximum reduction is more than −20% for approximately 5% of the systems which indicates that for most of the investigated systems (95%) the maximum reduction is less than 20%. The flooding efficiency and CSO efficiency are inversely proportional.

Only the bed shear stress performance is affected significantly by dry weather flow variations. A reduction of the dry weather flow by a factor smaller than 0.6 decreases the performance. In general, an increase produces only marginal improvements. The water demand reduction usually has no impact on the hydraulic pressure performance (WDS hydraulic). Conversely, an increase in the water demand leads to a pressure reduction because of increased friction losses. For the water quality, adequate performance can be assured even for a water demand of 60% provided that tank management is sufficient (filling and emptying cycles of the tanks), and that the demand reduction is equally distributed.

The WDS hydraulic performance (pressure performance) for small systems (class 1) is affected more compared to medium and large systems (classes 2 and 3). In particular, the small and medium systems reach their hydraulic limits earlier when increasing demands. In other words, the large systems are for the investigated scenarios more stable in terms of the demand increase because of their larger and redundant capacities. For the case study Innsbruck, a rapid drop in the hydraulic pressure performance is observed for increase factors above 1.4. This reduction is observed because the water resources in Innsbruck are (although available) not sufficiently exploited for such a high amount of water demand. The generated systems can have more capacity or can cover more demand because of the implemented simple design procedure which is solely based on economic flow velocities (for details see Sitzenfrei et al., 2013). For all classes, there is no impact in terms of the hydraulic performance when reducing the water demand. The water quality performance does not show any effects for water demand increases. For a water demand decrease, water quality reductions are visible as soon as the demand is reduced to 80% or less. In terms of transitions to, for instance, decentralised water recycling or water savings, the centralised urban drainage systems and water distribution system are sufficient until a reduction of −40% of the design water demand and dry weather flow, respectively.

**Fig. 10:**
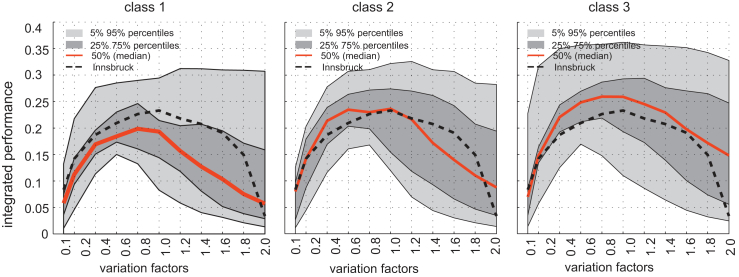
Integrated performance – stability of the networks.

Fig. 10 shows the integrated performance (multiplication of all performance indicators) against the different variation factors. The different performance indicators might be weighted differently, but for simplicity and to show the application, in this work they are weighted equally. The maximum values for classes 1 and 2 are comparable (95% percentile curve), but the 75% and 50% curves exhibit different behaviour. Comparing the different classes indicates that the small systems (class 1) are more likely to exhibit a lower integrated performance and to be (for the investigated scenarios) less stable in terms of the variations.

In class 2, only approximately 25% of the generated systems (over 75 percentile) are negligibly affected (variation ranges of 0.6 and 1.4). Although the case study Innsbruck has its best overall performance for the initial state (variation factor 1.0), the system is redundant and is not significantly affected for variation factors between 0.4 and 1.6. The large systems (class 3) are in general more likely to respond with a stable performance because of the applied variation factors.

## 6 Model building example with numerous virtual case studies

**Fig. 11:**
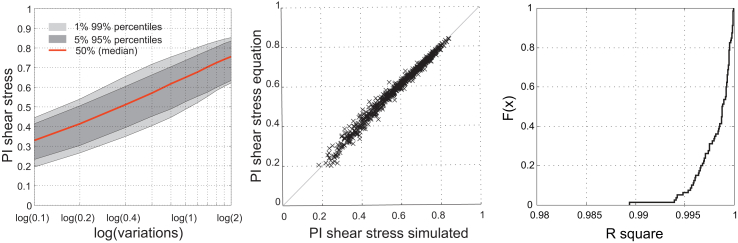
*PI* shear stress for the logarithm of the variations (left); regressions between the *PI* shear stress equation and simulation (middle); *R*^2^ for regressions from different starting points (right).

To note the benefit of using numerous virtual case studies for model building, the changes in the shear stress performance are discussed and interpreted in more detail. For the system performance indicator of the shear stress efficiency (UD shear stress), different variables, such as the hydraulic radius for different diameters and shapes and different slopes (see Equation 1), are taken into account. Likewise, the efficiency is influenced by the topology of the drainage network (e.g., network loops). However, the change in that performance indicator can be traced back to the change in the total dry weather flow production and therefore to the applied variation factors. In the graph on the left in Fig. 11, a base 10 logarithm is applied to all variation factors of the investigated systems. The 1, 5, 95 and 99 percentiles as well as the median value are shown. An almost linear coherence can be observed in that semi logarithmic plot.

In Equation 2, the regression is formulated for the observed data. With an observed or simulated performance indicator for the shear stress (*PI*_S,i_) and a corresponding total water flow in the combined urban drainage system (*Q*_i_), the performance indicator (*PI*_S,n_) for a changed dry weather flow production (*Q*_n_) can be predicted with Equation 2:

(2) $$PI_{\text{S,n}}=PI_{\text{S,i}}+{\text{log}}_{10}\frac{Q_{\text{n}}}{Q_{\text{i}}}0.3227\;\text{and}\;Q_{\text{n}}/Q_{\text{i}}\in \left[0.05,20\right]$$

*PI*_S,n_ calculated performance indicator for the shear stress for a total dry weather flow production in the system for *Q*_n_ (–); *PI*_S,n_ ∈ [0,1], *Q*_n_ ≠ 0, *Q*_i_ ≠ 0,

*PI*_S,i_ assessed performance indicator for the shear stress for a total dry weather flow production in the system for *Q*_i_ (–); *PI*_S,i_ ∈ [0,1], *Q*_i_ ≠ 0, *Q*_i_ ≠ 0.

For example, with an initial shear stress performance indicator of 0.7 (obtained value for the current situation *PI*_S,i_), a bisection of dry weather flow (*Q*_n_/*Q*_i_ = 0.5) reduces the shear stress performance to 0.6 (0.7 + log 10(0.5) × 0.3227). In the middle figure in Fig. 11, the results obtained with Equation 2 applied to the investigated systems are compared to the simulated results. For each of the 80 virtual case studies, in total 10 scenarios are tested (one marker represents such a scenario, resulting in 800 data points). Separately for each virtual case study, a regression analysis was performed.

In the figure on the right in Fig. 11, the cumulative distribution function of these 80 results for *R*^2^ is plotted. The median value of *R*^2^ for the 80 virtual cities is 0.998. All *R*^2^ values are above 0.988. Comparing the results from Equation 2 to the simulation results of the case study Innsbruck gives an *R*^2^ of 0.9904.

Solely with the results of the case study Innsbruck, Equation 2 could also have been developed. Although the results for the case study Innsbruck are also promising, Equation 2 would be very case specific and therefore less convincing as the analysis shown above.

## 7 Conclusions

The transition of traditional urban water systems towards more sustainable solutions has significant effects on the remaining central water networks. For a comprehensive assessment of the impact, it is necessary to investigate a large number of case studies. However, for modelling real-world systems, this is a very difficult undertaking because data collection, model building and calibration are tedious. In this paper, an alternative approach is presented and applied based on the stochastic generation of virtual case studies. VIBe (Virtual Infrastructure Benchmarking) is a tool that enables the stochastic generation of urban water systems for case study research with implemented data interfaces of external modelling software. An analysis of transition scenarios for water infrastructure based on these numerous case study data with varying characteristics leads to general, case-unspecific conclusions. In this work, an analysis of 80 virtual and one real-world case study is used to compare performance in systems of different sizes. Investigating the impact of the water demand decrease (e.g., due to water saving measures and transition in the implementation of decentralised water reuse systems) and water demand increase (e.g., population increase) on the water network reveals if adequate hydraulics and quality can be maintained in the network. Investigations related to numerous virtual systems reveal if the obtained results for the real-world system are outliers or a regular response to the investigated changes. Thus, percentages are determined to study how likely a system with a specific characteristic (e.g., population size) is to demonstrate representative behaviour. The percentages are determined based on evaluations of percentiles of performance assessments of many different systems. It is revealed that small systems (in context of this study defined as an Alpine system with a population between 70,000 and 100,000) are more affected as compared to medium (100,000–140,000) and large systems (140,000–170,000). In particular, large systems are for the investigated scenarios more stable in terms of the demand increase because of their larger and redundant capacities. Although the maximum values for small and medium systems are comparable, 75% of the small systems were identified to have a less stable integrated performance. A quarter of the medium systems are negligibly affected for variation ranges between 0.6 and 1.4. The investigated changes can represent transition scenarios over time. E.g., for the first decentralisation step there is a reduction factor of 0.8 applied and in second 0.6 can be regarded. This resembles a quasi-stationary consideration of dynamics. A time dependent change in the layout of the water networks (e.g., rehabilitation measures or network expansion) was not regarded here. Therefore, a representation of stepwise transition scenarios over a longer time horizon in the context of this paper is only applicable, as long as neglecting time dependent changes of the networks is appropriate.

Based on the analysis of 80 virtual and one real-world case study, a simple equation was derived to estimate the change in the shear stress performance due to changes in the dry weather flow production (water demand change). For the investigated systems, excellent agreements were obtained by comparing the complex, simulated and simplified, determined performances. Therefore, with the developed simplified approach, the shear stress performance can be predicted for dry weather flow changes for systems.
